# Clinical Outcomes of a Trifocal Versus an Extended Depth of Field Intraocular Lens in Chinese Patients With Cataract: A Prospective Cohort Study

**DOI:** 10.1155/2024/5571802

**Published:** 2024-10-15

**Authors:** Qingzhong Chen, Guangbin Zhang

**Affiliations:** ^1^Xiamen Eye Center and Eye Institute of Xiamen University, Xiamen, China; ^2^Xiamen Clinical Research Center for Eye Diseases, Xiamen, Fujian, China; ^3^Xiamen Key Laboratory of Ophthalmology, Xiamen, Fujian, China; ^4^Fujian Key Laboratory of Corneal & Ocular Surface Diseases, Xiamen, Fujian, China; ^5^Xiamen Key Laboratory of Corneal & Ocular Surface Diseases, Xiamen, Fujian, China; ^6^Translational Medicine Institute of Xiamen Eye Center of Xiamen University, Xiamen, Fujian, China

**Keywords:** cataracts, multifocal intraocular lenses, quality of life, visual acuity, visual disturbances

## Abstract

**Purpose:** To compare the standard outcomes between the PanOptix and Symfony multifocal intraocular lenses (MIOLs) in Chinese patients.

**Methods:** This prospective observational cohort study enrolled patients with cataracts between April 2021 and December 2021 at Xiamen Eye Center Affiliated to Xiamen University. The patients were grouped in the PanOptix (TIOL group) and Symfony (EDF group) according to the IOLs implanted. Uncorrected distant, corrected distance, binocular uncorrected intermediate, and near visual acuity (UNVA), distance-corrected intermediate and near VA (DCNVA) , defocus curve, spectacle independence, contrast sensitivity (CS), and visual disturbances were evaluated after 3 months.

**Results:** Forty patients (80 eyes) were enrolled in the study (20/group). Three months after the operation, UNVA (0.13 ± 0.16) and DCNVA (0.08 ± 0.08) were better in the TIOL group than in the EDF group (0.22 ± 0.08 and 0.22 ± 0.08, respectively, *p*=0.003 and *p* < 0.001, respectively). The TIOL group achieved a better-than-0.15-logMAR VA from +0.5 D to −2.5 D (40 cm). VAs of the TIOL group from −1 D (100 cm) to −4 D (25 cm) were better than in the EDF group (*p* < 0.05). There were no significant differences in the intermediate and far vision outcomes (*p* > 0.05). Total near-vision spectacle independence was higher in the TIOL group (80%) than in the EDF group (50%) (*p*=0.039).

**Conclusion:** Compared with EDF, TIOL leads to better near-vision outcomes without significant differences for intermediate and distant vision outcomes.

**Trial Registration:** ClinicalTrials.gov identifier: ChiCTR2100044558.

## 1. Introduction

The prevalence of cataracts is 5.6% in people aged 55–64 years and rapidly increases to 92.6% in people ≥80 years old [1]. Cataract surgery is indicated when visual function no longer meets the patient's far distance vision needs, and surgery is likely to improve vision [1, 2].

Monofocal intraocular lenses (IOLs) provide good distance vision, but most patients require reading glasses [3, 4]. Since cataracts are being diagnosed in ever younger patients who need to maintain productivity and daily activities, reading glasses might be inconvenient [5]. Fortunately, multifocal IOLs (MIOLs) can provide distance and near vision [6]. Patients who receive MIOLs have a better uncorrected near vision, improved overall quality of vision, and increased spectacle independence than those who receive monofocal IOLs [7, 8]. Nevertheless, the lenses had the disadvantage of reducing contrast sensitivity (CS) [6, 9]. Despite technological advances, many patients will have visual discomforts with MIOLs, including glares, halos, starbursts, and reduction of CS [5, 9–11]. However, understanding how these symptoms are affected by the cataract and the implanted MIOL can help to decide best surgery moment to avoid patient's perception of increasing these adverse events and better management of the balance with patient's expectations [12, 13].

The AcrySof IQ PanOptix TFNT00 (Alcon Laboratories, Inc., Fort Worth, TX, United States of America) is a trifocal MIOL designed to be more independent of pupil size [14–16]. Case series reported superior near, intermediate, and distance vision and improved CS and quality of life (QOL) with the PanOptix MIOL [17, 18]. The TECNIS Symfony ZXR00 lens (Johnson and Johnson Vision, Santa Ana, CA, United States of America) is an extended depth-of-focus (EDF) MIOL designed to provide an extended focus range by decreasing the overlapping of far and near images [19].

However, studies that compared the PanOptix versus Symfony MIOLs reported contradictory conclusions. A prospective study showed that the PanOptix had better postoperative best-corrected near defocus (−2.5D to −4D) compared with Symfony [20], while a retrospective study indicated that the PanOptix demonstrated an initial superiority at near distances compared with Symfony, but after 3 months, the two IOLs had similar outcomes [21]. Furthermore, the available studies on PanOptix versus Symfony were mainly focused on visual acuity (VA). For the assessment of subjective visual quality, previous studies mostly used the Visual Function Questionnaire (VFQ-25) [20] and the National Eye Institute Refractive Error Quality of Live Instrument (NEI-RQL) [22]. No studies used the Questionnaire for Visual Disturbances (QUVID), which includes questions about the patient's visual symptoms and experiences before and after IOL implantation for the reason that the questionnaire only could be used with the authorization of Alcon Inc. [23]. No previous study simultaneously compared multidimensional outcomes, including the whole range of visual outcomes, spectacle independence, CS, and visual disturbances between the PanOptix and Symfony MIOLs. Furthermore, no reports in the Chinese population compare those two MIOLs.

Therefore, this prospective cohort study aimed to compare the whole range of visual outcomes, spectacle independence, and visual disturbances between the PanOptix and TECNIS Symfony MIOLs in Chinese patients. The results could provide data for the rational selection of a MIOL in such patients.

## 2. Methods

### 2.1. Study Design and Patients

This prospective observational cohort study enrolled patients consecutively with cataracts between April 2021 and December 2021 in the Department of Ophthalmology at Xiamen Eye Center Affiliated to Xiamen University. The study was also approved by the Institutional Review Board of the Xiamen Eye Center affiliated to Xiamen University. The study was performed in accordance with the tenets of the Declaration of Helsinki. All participants provided written informed consent.

The inclusion criteria included those (1) aged ≥18 years, (2) diagnosed of nontraumatic cataract, and (3) with a desire to obtain excellent distance, intermediate, and near postoperative VA. The exclusion criteria included those (1) combined with other eye diseases which affect the visual outcome such as corneal dystrophy, glaucoma, macular degeneration, uveitis, diabetic retinopathy, retinal detachment, strabismus, and amblyopia; (2) with previous corneal or intraocular surgery; or (3) with corneal astigmatism more than 1 D, corneal higher-order aberrations, corneal spherical aberrations, and corneal coma over 0.3 microns, kappa angle over 0.5 mm, or any other factors affecting the effect of MIOL implantation.

### 2.2. Surgical Procedures and Grouping

This study adopted block randomization method for grouping. All participants underwent binocular cataract surgery and were grouped in the PanOptix IOL group (TIOL group) and Symfony IOL group (EDF group) according to the type of IOLs implanted. The surgery on the contralateral eye was done the day after the first eye, and the follow-up time was defined from the second eye surgery. The Barrett Universal II formula was used for IOL power calculation with IOL constants of 1.94 for PanOptix IOL [24] and 2.04 for Symfony IOL according to the IOL Con (https://iolcon.org/lensesTable.php).

All surgeries were performed by an experienced surgeon (Zhang GB). All patients received topical anesthesia using 0.5% proparacaine drops. A 2.2-mm main clear corneal incision and a second incision were made. The Alcon Centurion System was used for the phacoemulsification procedure. The MIOLs (PanOptix or Symfony) were implanted in the capsular bags. All the participants received binocular surgery and IOLs implantation within 2 weeks.

### 2.3. Assessments

Before the operation, slip lamp and ocular fundus examinations were performed to exclude anterior and posterior abnormalities. Uncorrected distant (5 m) VA (UDVA), best-corrected distant (5 m) VA (CDVA), subjective refraction, A-scan, and B-scan were performed and recorded before the surgery. Ocular biometry (Lenstar LS-900) was used to determine the power of the implanted IOLs. Scheimpflug corneal topography (Pentacam; Oculus Inc., Wetzlar, Germany) and OPD Scan (Nidek, Gamagori, Japan) were used to measure corneal astigmatism (CA), pupil size, corneal higher order aberrations, corneal spherical aberrations, corneal coma, kappa angle, and modulation transfer function (MTF).

Three months after binocular surgery, the participants were asked to return to the hospital for the postoperative evaluation. At the 3-month visit, UDVA, CDVA, subjective refraction, and intraocular pressure (IOP) were evaluated. In addition, binocular uncorrected intermediate VA (UIVA, 60 cm) and near VA (UNVA, 40 cm), distance-corrected intermediate VA (DCIVA, 60 cm) and near VA (DCNVA, 40 cm), defocus curve, MTF, spectacle independence, photopic and mesopic CS, and QUVID (translated into Chinese) [23] were evaluated 3 months postoperatively. Standardized logarithm of the minimum angle of resolution (logMAR) charts was used for VA measurement with photopic conditions defined as ∼85 cd/m^2^ and environmental illumination defined as inferior to 21.25 lux. The uncorrected and corrected near (40 cm), intermediate (60 cm), and far (4 m) binocular VA were measured using the reading table model of the Early Treatment Diabetic Retinopathy Study (ETDRS) charts. To obtain the defocus curve, binocular distant corrected VA was tested under photopic conditions (683 lm/W) and 100% contrast (ESV-3000 ETDRS System, Vector Vision, Inc.) at 4 m with additional lenses added sequenced over the range of +1.00 to −4.00 D in 0.50 D steps. To avoid memory effects, the letter sequence presented was random, and the patients' eyes were covered between the presentations of the lens, so the subjects did not know which lens was inserted and whether the letters on the chart were changed. Binocular distant–corrected CS under photopic and mesopic conditions was measured by using the CSV-1000 chart (Vector Vision, Inc., Greenville, OH, USA) at spatial frequencies of 0.5, 2, 5, 10, and 18 cycle per degree (cpd).

### 2.4. Statistical Analysis

The estimated sample size was calculated online (https://powerandsamplesize.com/Calculators/Compare-2-Means/2-Sample-Equality). The statistical analysis was performed using SPSS 23.0 (IBM, Armonk, New York, United States of America). The normality of data samples was evaluated using the Kolmogorov–Smirnov and Shapiro–Wilk tests. Continuous data with a normal distribution were expressed as means ± standard deviation and analyzed using the independent-sample *t*-test for intergroup differences and the paired-samples *t*-test for the pre-/postoperative comparisons. Data with a non-normal distribution were described by median (range) and analyzed using the Wilcoxon rank-sum test for intergroup differences and the Wilcoxon signed-rank test for the pre-/postoperative comparisons. Categorical data were presented as *n* (%) and analyzed using the chi-square test or Fisher's exact test. Two-sided *p* values < 0.05 were considered statistically significant.

## 3. Results

### 3.1. Preoperative Characteristics of the Patients

Forty patients were enrolled in the study, with 20 in the TIOL group (40 eyes) and 20 in the EDF group (40 eyes). There were no significant differences in age, axial length (AL), photopic pupil diameter (PPD), mesopic pupil diameter (MPD), anterior chamber depth (ACD), CA, mean keratometry (Km), UDVA, and CDVA between the TIOL and EDF groups before the operation (all *p*  > 0.05) (Table 1). All procedures were completed successfully without intraoperative complications.

### 3.2. VA

Three months after the operation, UNVA (0.13 ± 0.16) and DCNVA (0.08 ± 0.08) were significantly better in the TIOL group than in the EDF group (0.22 ± 0.08 and 0.22 ± 0.08 and *p*=0.003 and *p* < 0.001, respectively). On the other hand, UDVA, UIVA, CDVA, DCIVA, and MTF showed no significant changes between the two groups (all *p* > 0.05) (Table 2).

### 3.3. Binocular Defocus Curve

**Figure 1:**
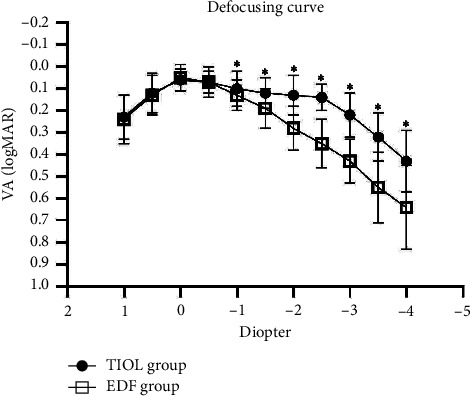
Binocular distance corrected defocus curves of the PanOptix IOL (TIOL) and Symfony IOL (EDF) groups 3 months after the operation. ^∗^*p* < 0.05, the error bars refer to standard deviation.

The results of the defocus curve showed that the TIOL group achieved a better-than-0.15-logMAR VA among the range from +0.5 D to −2.5 D (40 cm). VAs of the TIOL group from −1 D (100 cm) to −4 D (25 cm) were significantly better than in the EDF group (all *p* < 0.05) (Figure 1).

### 3.4. Spectacle Independence

The proportion of patients reporting never using spectacles for near vision was significantly higher in the TIOL group (80%) than in the EDF group (50%) at 3 months after the operation (*p*=0.039). The spectacle independence for far and intermediate vision showed no significant differences between the two groups at 3 months after the operation (all *p* > 0.05) (Table 3).

### 3.5. Photopic and Mesopic CS

**Figure 2:**
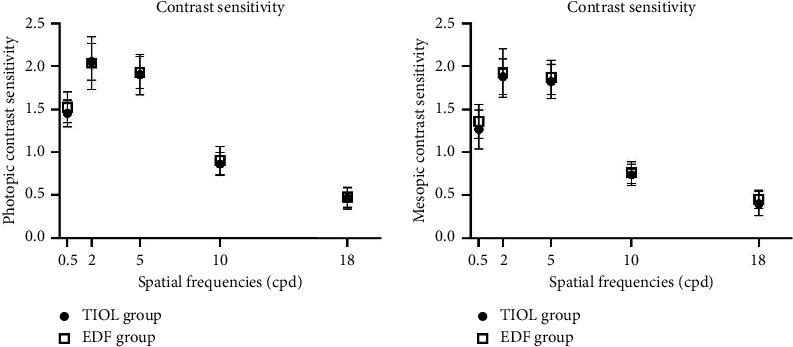
Binocular distance corrected photopic and mesopic CS of the PanOptix IOL (TIOL) and Symfony IOL (EDF) groups 3 months postoperatively. The error bars refer to standard deviation.

No significant differences were found in the photopic and mesopic CS between the two groups at the 0.5, 2, 5, 10, and 18 cpd levels at 3 months after the operation (all *p* > 0.05) (Figure 2).

### 3.6. Visual Disturbance

In the QUVID, > 75% of the patients in the TIOL group reported “not at all” or “a little bit” for starbursts, halos, and glares, which was comparable with the EDF group. No severe hazy vision, blurred vision, double vision, or dark areas were reported in the two groups (Table 4).

## 4. Discussion

This prospective cohort study compared the whole range of visual outcomes, spectacle independence, and visual disturbances between the PanOptix and Symfony MIOLs in Chinese patients. The results suggested that compared with the Symfony MIOL, the PanOptix MIOL leads to better near-vision outcomes without significant differences for intermediate and far vision outcomes. These results could help guide the selection of the proper MIOL according to the patient's needs.

To our knowledge, this study was the first to compare the TIOL and EDF IOL in Chinese patients with cataracts. Both IOLs showed satisfied VA outcomes, but TIOL provided better UNVA and DCNVA. It is generally well-accepted that different MIOLs achieve good intermediate and distant vision [20–22, 25, 26]. Indeed, both IOL types are designed for good distant vision [7, 8], but previous studies regarding the near vision between the TIOL and EDF IOL yielded contradictory results. Farvardin et al. [20] showed that the TIOL led to better UNVA than the EDF IOL but similar to DCNVA. Mencucci et al. [26] reported better UNVA with the TIOL than with the EDF IOL but did not report DCNVA. de Medeiros et al. [25], Mencucci et al. [26], and Pedrotti et al. [22] reported better UNVA and DCNVA with the TIOL than with the EDF IOL. On the other hand, Moshirfar et al. [21] reported no significant differences between the two MIOLs regarding postoperative UNVA but did not report DCNVA. The discrepancies among studies can be due to the populations of patients (e.g., age, ethnicity, postoperative refraction, and pupil size [27]). In addition, the study design (prospective versus retrospective) could also influence the outcomes.

The expected visual outcome over a distance range is an important surgical outcome. The defocus curve in the TIOL group was significantly better than in the EDF group, as supported by previous studies [20, 22, 25]. Previous studies also showed that multifocal and EDF lenses have defocus curves with high acuity across near, intermediate, and distance [28, 29].

Near-vision spectacle independence in the TIOL group was higher than in the EDF group, without significant differences for intermediate and distant vision, as supported by the literature [22]. A previous study even showed a 98% rate of spectacle independence with a TIOL [30]. The rate of near-vision spectacle independence was 80% in the present study, lower than 98% in the previous study [30], but the differences could be due to the age of the patients, their occupation, and their need for near VA, among others.

Although EDF is designed to have an improved CS [19], this study showed no significant differences regarding photopic CS between the two groups. Nevertheless, previous studies showed better CS with the EDF IOL than with the TIOL [25, 26].

A main issue with MIOLs compared with monofocal IOLs is the higher incidence of photic phenomena [31]. Although neuroadaptation can occur [32], not all patients can achieve a similar level of neuroadaptation [33]. Although neuroadaptation was not directly evaluated in the present and previous studies, patient-reported outcomes (PROMs) can be used as a surrogate to determine how patients cope with their new vision. This study was the first to use the QUVID in a Chinese population. Previous studies used NEIVFQ-25 [30], the VFQ-25 [20], or the NEI-RQL [22] to assess the visual disturbances after TIOL or EDF IOL implantation. Taking into account, the PROMs is important for the management of cataracts [34, 35], and the American Academy of Ophthalmology (AAO) and the United States Food and Drug Administration (FDA) Task Force on premium IOLs stated that the available PROM tools were inadequate for visual symptoms after IOL implantation [36]. QUVID is a new tool that provides information about the patient's visual symptoms and experiences before and after IOL implantation [23].

The common visual disturbances in the two groups were starbursts, halo, and glare, which were all commonly seen after IOL implantation [37]. The visual symptoms were also similar between the two groups. Teshigawara et al. [27] reported that halo size was larger with the TIOL, while halo intensity was higher with the EDF IOL. Moshirfar et al. [38] reported earlier visual symptoms with the EDF IOL than with the TIOL.

This study had limitations. There was only one postoperative time point (3 months), and the follow-up was short. Although the visual outcomes of patients tend to be basically stable 3 months after surgery, further study should make multiple postoperative assessments at varying intervals, such as 1 month, 6 months, and 12 months, to obtain a more comprehensive understanding of both short-term and long-term postoperative changes.

In conclusion, the PanOptix TIOL leads to better near-vision outcomes than the Symfony EDF, including UNVA, DCNVA, a higher rate of near-vision spectacle independence, and a better-than-0.15-LogMAR VA among the range from +0.5 D to −2.5 D (40 cm) but without significant differences in UDVA, UIVA, CDVA, DCIVA, MTF, photopic CS, or visual disturbances. These results could help guide the selection of the proper MIOL according to the patient's needs.

## Figures and Tables

**Table 1:** Baseline characteristics of the participants.

	TIOL	EDF	*P*
Age, years			
Mean ± sd	61.70 ± 14.70	57.75 ± 14.18	0.392
Median	66	54	
Min, max	(32, 77)	(33, 82)	
IQR	(52.75, 72)	(47, 68.25)	
AL			
Mean ± sd	23.70 ± 0.97	24.02 ± 1.17	0.186
Median	23.50	23.83	
Min, max	(22.12, 26.22)	(22.36, 27.54)	
IQR	(23.01, 24.11)	(23.34, 24.39)	
CA			
Mean ± sd	0.52 ± 0.22	0.53 ± 0.34	0.803
Median	0.55	0.48	
Min, max	(0, 1.02)	(0.10, 1.50)	
IQR	(0.31, 0.68)	(0.26, 0.70)	
ACD			
Mean ± sd	3.20 ± 0.23	3.17 ± 0.25	0.560
Median	3.25	3.21	
Min, max	(2.68, 3.56)	(2.68, 3.59)	
IQR	(3.00, 3.36)	(3.01, 3.33)	
Km			
Mean ± sd	43.69 ± 1.16	43.53 ± 1.15	0.541
Median	43.65	43.44	
Min, max	(41.79, 45.87)	(41.28, 45.84)	
IQR	(42.84, 44.72)	(42.87, 44.39)	
PPD			
Mean ± sd	3.05 ± 0.32	3.08 ± 0.23	0.591
Median	3.05	3.05	
Min, max	(2.49, 3.77)	(2.66, 3.58)	
IQR	(2.76, 3.29)	(2.88, 3.25)	
MPD			
Mean ± sd	4.72 ± 0.42	4.81 ± 0.33	0.290
Median	4.73	4.78	
Min, max	(4.02, 5.46)	(4.22, 5.47)	
IQR	(4.30, 5.03)	(4.58, 5.02)	
IOL power			
Mean ± sd	20.98 ± 2.00	21.18 ± 1.97	0.653
Median	21.00	21.00	
Min, max	(17.00, 24.50)	(17.50, 25.00)	
IQR	(19.5, 22.88)	(19.5, 22.50)	
UDVA			
Mean ± sd	0.73 ± 0.38	0.74 ± 0.36	0.904
Median	0.70	0.70	
Min, max	(0.30, 1.50)	(0.20, 1.70)	
IQR	(0.33, 1.00)	(0.50, 1.00)	
CDVA			
Mean ± sd	0.40 ± 0.25	0.39 ± 0.25	0.823
Median	0.30	0.40	
Min, max	(0, 1.00)	(0, 1.20)	
IQR	(0.20, 0.50)	(0.20, 0.50)	

Abbreviations: ACD, anterior chamber depth; AL, axial length; CA, corneal astigmatism; CDVA, best-corrected distant (5 m) VA; EDF, Symfony IOL; Km, mean keratometry; MPD, mesopic pupil diameter; PPD, photopic pupil diameter; TIOL, PanOptix IOL; UDVA, uncorrected distant (5 m) VA.

**Table 2:** Binocular VA outcomes 3 months after the operation.

	TIOL	EDF	*P*
UDVA	0.07 ± 0.05	0.06 ± 0.07	0.570
UIVA-60 cm	−0.03 ± 0.07	−0.01 ± 0.08	0.104
UNVA-40 cm	0.13 ± 0.16	0.22 ± 0.08	0.003
CDVA	0.01 ± 0.02	0.02 ± 0.04	0.140
DCIVA-60 cm	−0.05 ± 0.04	−0.02 ± 0.06	0.127
DCNVA-40 cm	0.08 ± 0.08	0.22 ± 0.08	< 0.001
Refraction	−0.08 ± 0.21	−0.11 ± 0.21	0.541

Abbreviations: CDVA, binocular best-corrected distant (5 m) VA; DCIVA, binocular distance-corrected intermediate VA; DCNVA, binocular distance-corrected near VA; EDF, Symfony IOL; TIOL, PanOptix IOL; UDVA, binocular uncorrected distant (5 m) VA; UIVA, binocular uncorrected intermediate VA; UNVA, binocular uncorrected near VA.

**Table 3:** Spectacle independence of the two groups 3 months after the operation.

	TIOL	EDF	*P*
For distant vision			/
Never	20 (100%)	20 (100%)	
Sometimes	0	0	
Always	0	0	
For intermediate vision			0.376
Never	18 (90%)	16 (80%)	
Sometimes	2 (10%)	4 (20%)	
Always	0	0	
For near vision			0.039
Never	16 (80%)	10 (50%)	
Sometimes	4 (20%)	5 (25%)	
Always	0	5 (25%)	

Abbreviations: EDF, Symfony IOL; TIOL, PanOptix IOL.

**Table 4:** QUVID questionnaire of the TIOL and EDF groups 3 months postoperatively.

	TIOL group (*n* = 20), *n* (%)					EDF group (*n* = 20), *n* (%)				
	Not at all	A little bit	Somewhat	Quite a bit	Very much	Not at all	A little bit	Somewhat	Quite a bit	Very much
Starbursts	10 (50%)	5 (25%)	2 (10%)	2 (10%)	1 (5%)	11 (55%)	4 (20%)	2 (10%)	2 (10%)	1 (5%)
Halo	11 (55%)	4 (20%)	4 (20%)	0	1 (5%)	11 (55%)	3 (15%)	2 (10%)	2 (10%)	2 (10%)
Glare	12 (60%)	5 (25%)	1 (5%)	1 (5%)	1 (5%)	12 (60%)	4 (20%)	2 (10%)	1 (5%)	1 (5%)
Hazy vision	15 (75%)	4 (20%)	1 (5%)	0	0	14 (70%)	4 (20%)	2 (10%)	0	0
Blurred vision	15 (75%)	4 (20%)	1 (5%)	0	0	16 (80%)	3 (15%)	1 (5%)	0	0
Double vision	18 (90%)	2 (10%)	0	0	0	18 (90%)	2 (10%)	0	0	0
Dark area	16 (80%)	3 (15%)	1 (5%)	0	0	17 (85%)	2 (10%)	1 (5%)	0	0

*Note:* “Not at all” is referred to free of visual disturbances; “A little bit” and “Somewhat” are referred to minor visual disturbances; “Quite a bit” and “Very much” are referred to moderate–severe visual disturbances that make bothersome.

Abbreviations: EDF, Symfony IOL; TIOL, PanOptix IOL.

## Data Availability

The data used to support the findings of this study are available from the corresponding author upon request. The data are not publicly available due to privacy or ethical restrictions.
